# Phosphatidylethanolamine-binding protein 4 deficiency exacerbates carbon tetrachloride-induced liver fibrosis by regulating the NF-κB signaling pathway

**DOI:** 10.3389/fphar.2022.964829

**Published:** 2022-09-02

**Authors:** Qianqian Luo, Yuanyi Ling, Yufei Li, Xiaoqin Qu, Qiaoqing Shi, Shuangyan Zheng, Yanhong Li, Yonghong Huang, Xiaoyan Zhou

**Affiliations:** ^1^ Department of Pathophysiology, Medical College of Nanchang University, Nanchang, China; ^2^ Nanchang Joint Program, Queen Mary School, Medical College of Nanchang University, Nanchang, China; ^3^ The Center of Laboratory Animal Science, Nanchang University, Nanchang, China; ^4^ Department of Forensic Medicine, Medical College of Nanchang University, Nanchang, China; ^5^ Jiangxi Province Key Laboratory of Tumor Etiology and Molecular Pathology, Nanchang, China

**Keywords:** PEBP4, liver fibrosis, NF-κB, mouse model, signaling pathway

## Abstract

Liver fibrosis is a pathological process which can progress to hepatocirrhosis, even hepatocellular carcinoma. Phosphatidylethanolamine-binding protein 4 (PEBP4) is a secreted protein involved in regulating many molecular pathways, whereas its roles in diseases including hepatic fibrosis remain undefined. The nuclear factor-κappa B (NF-κB) signaling pathway has been found to be involved in the development of liver fibrosis. In this study, we generated a hepatocyte-conditional knockout (CKO) mouse model of PEBP4, and explored the potential functions of PEBP4 on liver fibrosis and the NF-κB signaling pathway in a mouse model of carbon tetrachloride (CCl4)-induced liver fibrosis. We demonstrated that PEBP4 CKO aggravated CCl4-triggered liver fibrosis, as evidenced by altered histopathology, an increase in the activities of alanine aminotransferase (ALT), aspartate aminotransferase (AST) and hydroxyproline (HYP) levels, and more collagen deposition, as well as by enhanced expression of fibrotic markers including α-smooth muscle actin (α-SMA), collagen I and collagen III. Mechanistically, PEBP4 deficiency activated the NF-κB signaling pathway, as indicated by increased phosphorylation of NF-κB p65 and inhibitor protein κB inhibitor-α (IκB-α), and nuclear NF-κB p65 expression in the fibrotic liver. Notably, the NF-κB inhibitor pyrrolidine dithiocarbamate (PDTC) partially blocked the activation of the NF-κB pathway, and reversed the pro-fibrotic effect of PEBP4 deletion in CCl4-treated mice. Together, these results suggest that PEBP4 deficiency results in aggravation of liver fibrosis and activation of the NF-κB signaling pathway, supporting a novel concept that PEBP4 is a crucial player in hepatic fibrosis, but also might be a negative regulator of the NF-κB signaling in liver fibrosis.

## Introduction

Liver fibrosis is a reversible wound healing response to hepatic injury and inflammation, characterized by excessive deposition of extracellular matrix (ECM) proteins (Friedman, 2003). Sustained liver damage drives fibrosis to progress into cirrhosis, an irreversible hepatic disease with poor prognosis and high mortality (Albanis and Friedman, 2001; Hernandez-Gea and Friedman, 2011). It is well-known that hepatic stellate cells (HSCs) are responsible for ECM synthesis and remodeling, and activated HSCs are the major source of hepatic myofibroblasts in liver fibrosis. Chronic liver injury stimulates the activation of HSCs, subsequently leading to the accumulation of ECM, especially collagen I and collagen III (Friedman, 2003; Gäbele et al., 2003). In healthy liver, HSCs are located in the space of Disse, manifesting a quiescent phenotype. Responding to chronic injury, HSCs become activated and transdifferentiate into myofibroblast-like cells. These myofibroblasts then migrate to sites of injury and secret ECM, thereby forming fibrous scars (Kisseleva and Brenner, 2021). Accordingly, the progression of liver fibrosis can be identified by specific markers for hepatic myofibroblasts. For instance, α-smooth muscle actin (α-SMA) is generally employed to characterize advance fibrosis and mark hepatic myofibroblasts, so that it is considered as an important marker of collagen accumulation and scar formation in liver fibrosis (Lan et al., 2018; Kisseleva and Brenner, 2021).

Phosphatidylethanolamine-binding protein 4 (PEBP4) is a newly discovered secreted protein of the PEBP family, and is expressed in multiple human tissues such as the heart, lung, colon, prostate, and liver (He et al., 2016; Luo et al., 2019). Existing studies have revealed the effects of PEBP4 on multiple human cancers. In breast cancer, PEBP4 silencing can promote tumor necrosis factor-α (TNF-α)-induced apoptosis by activating mitogen-activated protein kinase (MAPK) signaling cascade (Wang et al., 2005), and suppress proliferation and invasion by repressing phosphoinositide 3-kinase (PI3K)/Akt pathway (Wang et al., 2017). Similarly, PEBP4 knockdown can activate extracellular signal-regulated kinase 1/2 (ERK1/2) pathway, thereby inhibiting proliferation and migration in glioma cells (Huang et al., 2019), whereas its overexpression can potentiate tumor growth by stimulating PI3K/Akt/mTOR pathway in non-small cell lung cancer (NSCLC) (Yu et al., 2015). In addition, PEBP4 is reported to be involved in the activation of sonic hedgehog (SHH) pathway (Jian et al., 2019). These research indicate that PEBP4 is potentially involved in many molecular pathways to modulate tumor pathogenesis (Luo et al., 2019), but its potential role in other diseases like hepatic fibrosis has not yet been discussed.

Nuclear factor-κappa B (NF-κB) has been found to play a role in hepatic injury and fibrosis *via* interfering with the function of hepatocyte and HSC (Inokuchi et al., 2010). For instance, NF-κB is activated in most chronic liver diseases, and regulates hepatocyte apoptosis and HSC activation. It has been reported that NF-κB participates in liver fibrogenesis by regulating hepatic injury, inflammatory signals and the fibrogenic responses of HSCs (Luedde and Schwabe, 2011).

In this study, we demonstrated the antifibrotic roles of PEBP4 by combining hepatocyte-specific PEBP4 knockout with carbon tetrachloride (CCl4)-induced liver fibrosis mice. Mechanistically, we found that the deletion of PEBP4 activated the NF-κB signaling pathway. Furthermore, the negative regulation of PEBP4 on the NF-κB signaling and fibrogenesis in the liver was partially reversed by the NF-κB inhibitor pyrrolidine dithiocarbamate (PDTC). Taken together, our findings indicate that PEBP4 deficiency aggravates liver fibrosis and activates the NF-κB signaling pathway, providing a novel insight into the roles of PEBP4 in diseases.

## Materials and methods

### Animals

C57BL/6N wild type (WT) mice were purchased from GemPharmatech (Jiangsu, China). The *PEBP4* ^*flox/+*^ mice and the albumin (Alb)-Cre^+^ mice with C57BL/6N background were obtained from Cyagen Biosciences (Jiangsu, China). All mice were housed in a pathogen-free animal facility with free access to food and water. All animal experimental procedures were conducted according to the National Institutes of Health Guide for the Care and Use of Laboratory Animals, and the protocols approved by the Animal Care and Use Committee of Nanchang University.

### Generation and identification of phosphatidylethanolamine-binding protein 4 conditional knockout mice

PEBP4 CKO mice were generated by cross breeding. Firstly, *PEBP4* ^*flox/flox*^ mice were produced by crossing *PEBP4* ^*flox/+*^ mice with each other. Then *PEBP4* ^*flox/flox*^ mice were crossed with Alb-Cre^+^ mice to generate *PEBP4* ^*flox/+*^, *Alb-Cre* ^*+*^ mice. Finally, *PEBP4* ^*flox/flox*^, *Alb-Cre* ^*+*^ mice, namely PEBP4 CKO mice, were generated by mating *PEBP4* ^*flox/+*^, *Alb-Cre* ^*+*^ mice with each other.

Tail and liver DNAs from different mice were employed to analyze their genotypes. The genotypes were identified by polymerase chain reaction (PCR) analysis. The details about primers and their corresponding products in these PCR assays were described in Table 1. Agarose gel electrophoresis was applied to separate the PCR products.

**TABLE 1:** Sequence used for PCR.

**No.**	**Primer name**	**Primer sequence (5′-3′)**	**Band size**
P1	loxP F	GAT​CCT​GGA​GCT​ACT​GAA​AGC​ACT​GAG	flox = 251 bp
	loxP R	GCT​ATT​TAC​ACC​ACC​ATG​CCC​TGC	WT = 188 bp
P2	Alb-Cre F	GAA​GCA​GAA​GCT​TAG​GAA​GAT​GG	Alb-Cre = 390 bp
	Alb-Cre R	TTG​GCC​CCT​TAC​CAT​AAC​TG	
P3	PEBP4 del-F	GAT​CCT​GGA​GCT​ACT​GAA​AGC​ACT​GAG	PEBP4 KO = 277 bp
	PEBP4 del-R	ACA​ACC​AGA​AGG​ATG​AAA​TCG​GAA​AC	
P4	GAPDH F	TGG​ATT​TGG​ACG​CAT​TGG​TC	GAPDH = 251 bp
	GAPDH R	TTT​GCA​CTG​GTA​CGT​GTT​GAT	

DNA samples from the heart, liver, spleen, lung, and kidney of WT mice and CKO mice were collected. Then PEBP4 expression was detected by PCR and agarose gel electrophoresis. Glyceraldehyde-3-phosphate dehydrogenase (GAPDH) was used for PCR loading control. Primer sequences are listed in Table 1.

### Liver fibrosis model

For the experiment of CCl4 treatment alone, WT mice and PEBP4 CKO mice (aged 6–8 weeks, weighing 18–22 g) were randomly divided into four groups (*n* = 10 per group), i.e., WT control group, WT CCl4 group, CKO control group and CKO CCl4 group. Mice model with liver fibrosis were generated as described previously. Briefly, mice in CCl4 groups were intraperitoneally injected with 20% CCl4 to induce liver fibrosis for 8 weeks, twice per week. Mice in the control groups were injected with equal amount of olive oil. The blood and liver tissues were collected from each group 2 weeks after drug discontinuance.

For the experiment of PDTC combined with CCl4 treatment, WT mice and PEBP4 CKO mice were randomly divided into four groups (*n* = 10 per group): WT CCl4 group, WT CCl4 + PDTC group, CKO CCl4 group and CKO CCl4 + PDTC group. Mice in all groups were intraperitoneally injected with 20% CCl4, and mice in the PDTC groups received 60 mg/kg of PDTC, followed by CCl4 injection after 30 min. All administrations were given twice a week for 8 weeks. After 2 weeks of withdrawal, the blood and liver tissues were sampled from each group.

### Histological staining

Liver tissues were fixed with 4% paraformaldehyde, embedded in paraffin and sectioned at 4–6 μm for histological analysis. Samples were deparaffinized and rehydrated prior to staining. Hematoxylin and eosin (HE) and Masson staining were performed following standard procedures using HE and Masson dye solution kits (Servicebio, China). Morphological changes were evaluated under light microscope (Olympus Corporation, Tokyo, Japan).

### Evaluation of liver function and collagen content

The activities of serum alanine transaminase (ALT) and aspartate transaminase (AST), important indicators of liver function, were measured using a commercial kit (Nanjing Jiancheng Bioengineering Institute, China). The activity of hydroxyproline (HYP), reflecting the collagen concentration, were determined with an HYP assay kit (Nanjing Jiancheng Bioengineering Institute, China).

### Western blotting analysis

Total proteins were extracted from liver tissues and protein concentrations were determined by BCA protein assay kit (Beyotime Biotechnology, China) following the manufacturer’s protocol. Nuclear proteins were extracted using nuclear protein extraction kit (Bestbio, China). Proteins were separated by 10% sodium dodecyl sulfate polyacrylamide gel electrophoresis (SDS-PAGE) and then transferred to polyvinylidene fluoride (PVDF) membranes. PVDF membranes containing proteins were blocked with 5% skim milk in TBST buffer, and subsequently incubated with primary antibodies overnight at 4°C. Membranes with phosphorylated proteins were blocked with 5% BSA in TBST, followed by primary antibody incubation as well. Primary antibodies were specific against α-SMA (1:5000; Abcam), GAPDH (1:1000; Proteintech), collagen I (1:1000; Proteintech), collagen III (1:1000; Proteintech), NF-κB p65 (1:1000; Proteintech), Lamin B1 (1:1000; Proteintech), p-NF-κB P65 (1:1000; Cell Signaling Technology), p-inhibitor protein κB inhibitor (IκB)-α (1:1000; Cell Signaling Technology) and IκB-α (1:1000; Cell Signaling Technology). The membranes were incubated with the corresponding horseradish peroxidase-conjugated secondary antibodies (1:5000) the next day at room temperature for 2 h. Gels were visualized with a gel imaging system (Bio-Rad, United States) and performed quantitative analysis using the ImageJ software (National Institutes of Health, United States).

### Statistical analysis

All data in this study were presented as mean ± standard deviation (SD). Student’s *t*-test was conducted for comparison between two groups. One-way analysis of variance (ANOVA) was used to statistically compare differences among multiple groups using SPSS version 26.0 (IBM Corporation, United States). Plots were generated in GraphPad Prism version 8.0 (GraphPad Software, United States). *p* < 0.05 was considered statistically significant.

## Results

### Hepatocyte-specific phosphatidylethanolamine-binding protein 4 knockout mice are successfully generated

**FIGURE 1:**
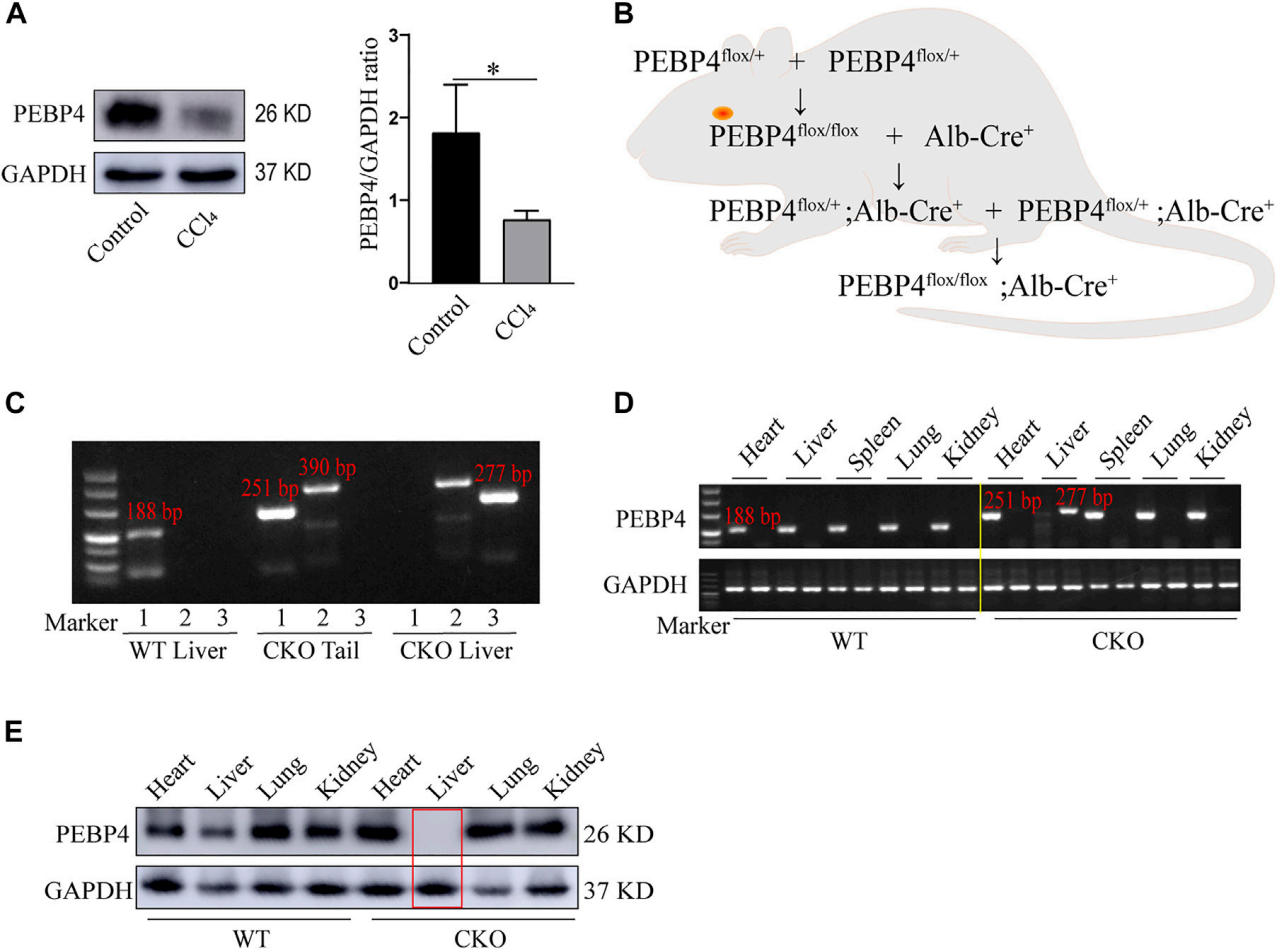
Construction and identification of PEBP4 hepatocyte-conditional knockout (CKO) mice. **(A)** Western blotting demonstrated that PEBP4 was downregulated in the liver tissue of WT CCl4 mice compared with the WT control mice. GAPDH was used as a loading control. **(B)** Schematic diagram of PEBP4 CKO mice (*PEBP4* ^*flox/flox*^ *, Alb-Cre* ^*+*^ mice) construction using the breeding technique. **(C)** Genotyping of mice by PCR analysis. The loxP sites (lane 1), the Alb-Cre transgene (lane 2), and PEBP4 allele (lane 3) of tail DNA and liver DNA were detected. **(D)** The loxP sites (lane 1) and PEBP4 allele (lane 2) in different tissues of WT and CKO mice were detected by PCR and agarose gel electrophoresis. **(E)** PEBP4 protein expression in different tissues of WT and CKO mice was detected by western blotting. Data are expressed as mean ± SD. *p < 0.05 vs. WT mice in the control group (Student’s *t*-test).

To investigate the association between PEBP4 expression and liver fibrosis, we firstly measured the protein levels of PEBP4 in control and CCl4-treated WT mice by western blotting. Results showed that PEBP4 expression was significantly decreased in CCl4-stimulated mice (Figure 1A). To explore the role of PEBP4 in liver fibrosis, we then obtained PEBP4 CKO mice by breeding technique, and the breeding process was outlined in Figure 1B. Agarose gel electrophoresis was applied to verify the genotypes of transgenic mice (Figure 1C) and the conditional knockout of PEBP4 in liver (Figure 1D). Also, western blotting demonstrated that PEBP4 deletion was specifically present in the liver of the CKO mice, and not in the liver of the WT mice or in the heart, spleen, lung and kidney of both the WT and CKO mice (Figure 1E). Thus, these results show that PEBP4 CKO mice are successfully constructed.

### Loss of phosphatidylethanolamine-binding protein 4 aggravates carbon tetrachloride-Induced liver fibrosis

**FIGURE 2:**
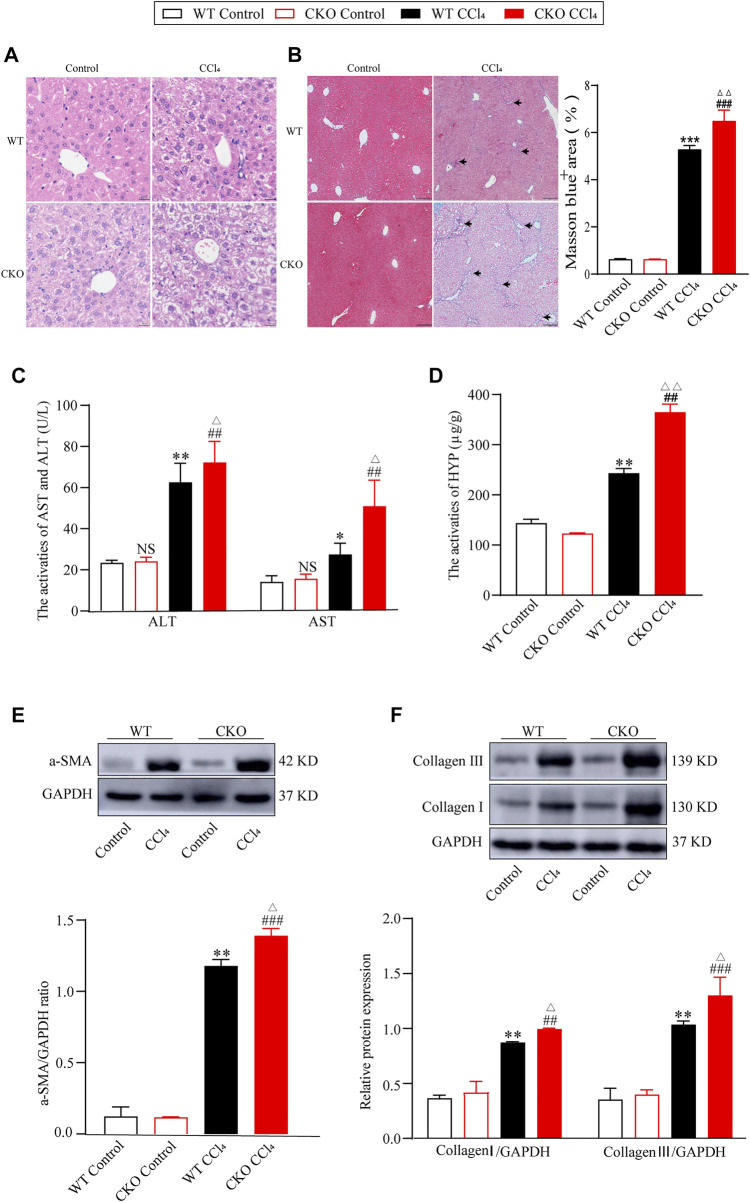
PEPB4 CKO aggravates CCl4-induced liver fibrosis. **(A)** Histological changes of liver tissues by HE staining (×200 magnification). **(B)** Collagen deposition of liver tissues by Masson staining (×100 magnification) and its quantitative analysis. Arrowhead indicates accumulated collagen. **(C)** The AST and ALT activities in the serum of mice (*n* = 6). **(D)** The HYP activity in the serum of mice (*n* = 6). **(E)** The protein expression of α-SMA was detected by western blotting (*n* = 3). **(F)** The protein expressions of collagen I and collagen III were detected by western blotting (*n* = 3). Data are expressed as mean ± SD. *p < 0.05, **p < 0.01 and ***p < 0.001 vs. the WT control group. ^##^ *p* < 0.01 and ^###^ *p* < 0.001 vs. the CKO control group. ^Δ^ *P* < 0.05 and ^ΔΔ^ *P* < 0.01 vs. the WT CCl4 group. NS, *p* > 0.05 not significant (ANOVA Test).

On the basis of the decrease in PEBP4 expression in fibrotic liver, we then detected its role in hepatic fibrosis in PEBP4 CKO mice subjected to CCl4. Histologically, HE staining revealed that PEBP4 deficiency aggravated CCl4-induced disorganized liver structure with steatosis as compared with the control groups (Figure 2A). More importantly, Masson staining indicated that CCl4 treatment led to collagen deposition both in the PEBP4 CKO and in the WT mice. Furthermore, the collagen accumulation in the PEBP4 CKO mice became more severe than that in the WT mice (*p* < 0.01) (Figure 2B). In addition, activities of ALT and AST were increased noticeably in the WT and CKO CCl4 groups, and CCl4-treated CKO mice showed a more significant rise (*p* < 0.05), suggesting an exacerbation of liver fibrotic damage (Figure 2C). Consistently, HYP activities in CCl4-induced groups were considerably higher compared with control groups, and the CKO CCl4 mice displayed a more striking rise (*p* < 0.01) (Figure 2D). Similarly, loss of PEBP4 notably enhanced the upregulation of α-SMA, collagen I and collagen III, the most abundant ECM proteins in fibrotic liver, caused by CCl4 treatment (Figures 2E,F). What’s more, in experimental groups, PEBP4 knockout resulted in higher expression of these ECM markers (*p* < 0.05). Altogether, all of these results demonstrate that PEBP4 plays a crucial role in the fibrogenesis of liver.

### Phosphatidylethanolamine-binding protein 4 knockout activates the NF-κB signaling pathway

**FIGURE 3:**
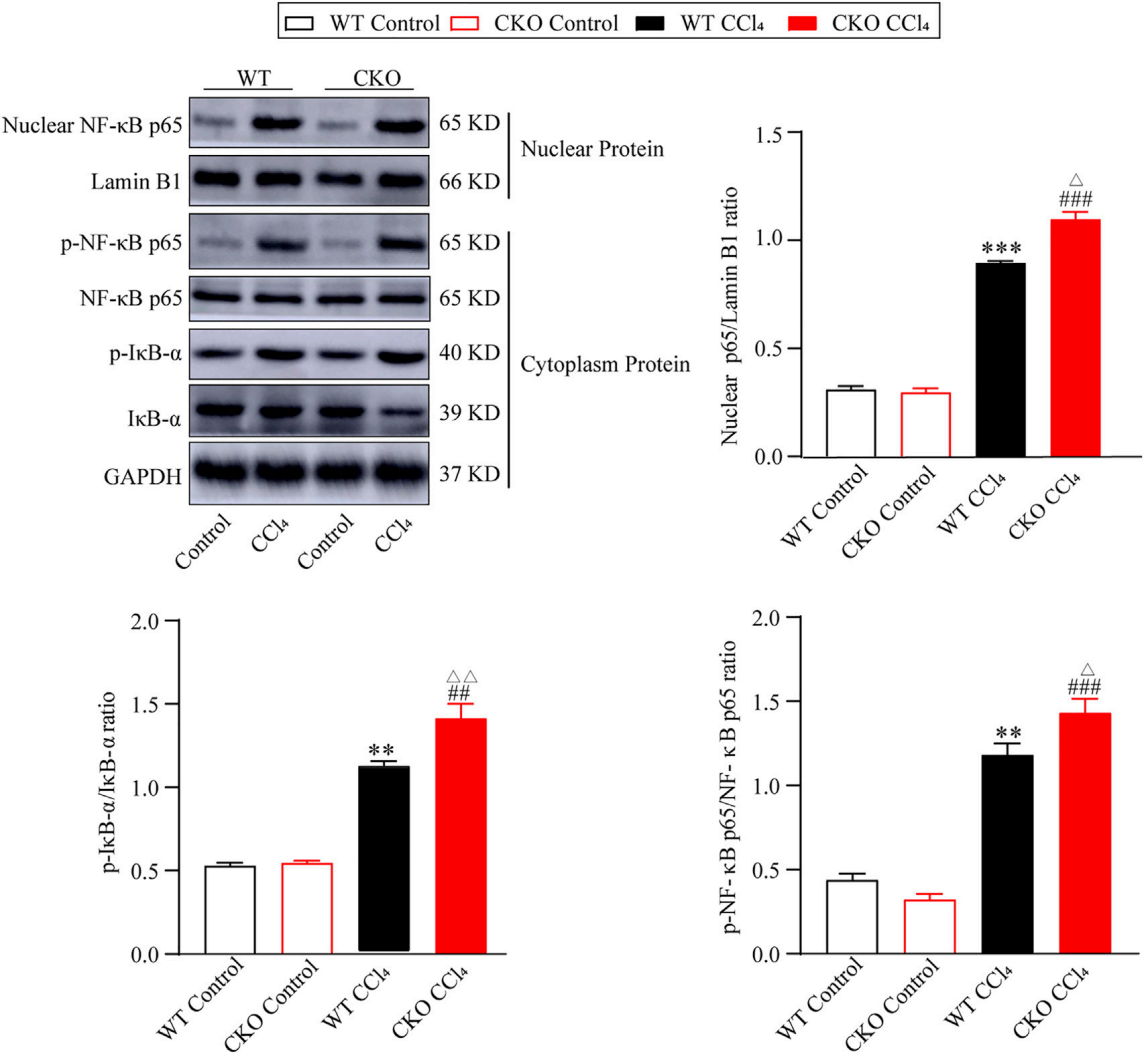
PEBP4 knockout activates NF-κB pathway. Protein expressions of nuclear NF-κB p65, p-NF-κB p65 and p-IκB-α were measured by western blotting, and Lamin B1 and GAPDH were applied as internal references of the nuclear and total protein. Data are expressed as mean ± SD. **p < 0.01 and ***p < 0.001 vs. the WT control group. ^##^ *p* < 0.01 and ^###^ *p* < 0.001 vs. the CKO control group. ^Δ^ *P* < 0.05 and ^ΔΔ^ *P* < 0.01 vs. the WT CCl4 group (ANOVA Test).

The NF-κB signaling pathway was reported to play a vital role in liver fibrosis. To define the effect of PEBP4 on the NF-κB pathway in hepatic fibrosis, we performed the protein expression of the NF-κB pathway by western blotting assays. As shown in Figure 3, the protein expression of nuclear NF-κB p65 in CCl4-treated groups was higher than that in control groups (*p* < 0.001), and its expression in the CKO CCl4 group increased more significantly (*p* < 0.05). Likewise, the p-IκB-α/IκB-α ratio, indicating the phosphorylated level of IκB-α, was considerably higher in experimental groups (*p* < 0.01), and the CKO CCl4 group showed a more noticeable rise (*p* < 0.01). Consistently, compared with control groups, the p-NF-κB p65/NF-κB p65 ratio was increased in the WT CCl4 group (*p* < 0.01) and CKO CCl4 group (*p* < 0.001). Moreover, the phosphorylation of NF-κB p65 was increased more significantly in the CKO CCl4 group (*p* < 0.05). Thus, these data imply PEBP4 knockout results in the activation of the NF-κB signaling in the liver fibrosis model.

### Pyrrolidine dithiocarbamate partially attenuates the effect of phosphatidylethanolamine-binding protein 4 deletion on liver fibrosis *via* the nuclear factor-κappa B signaling pathway

**FIGURE 4:**
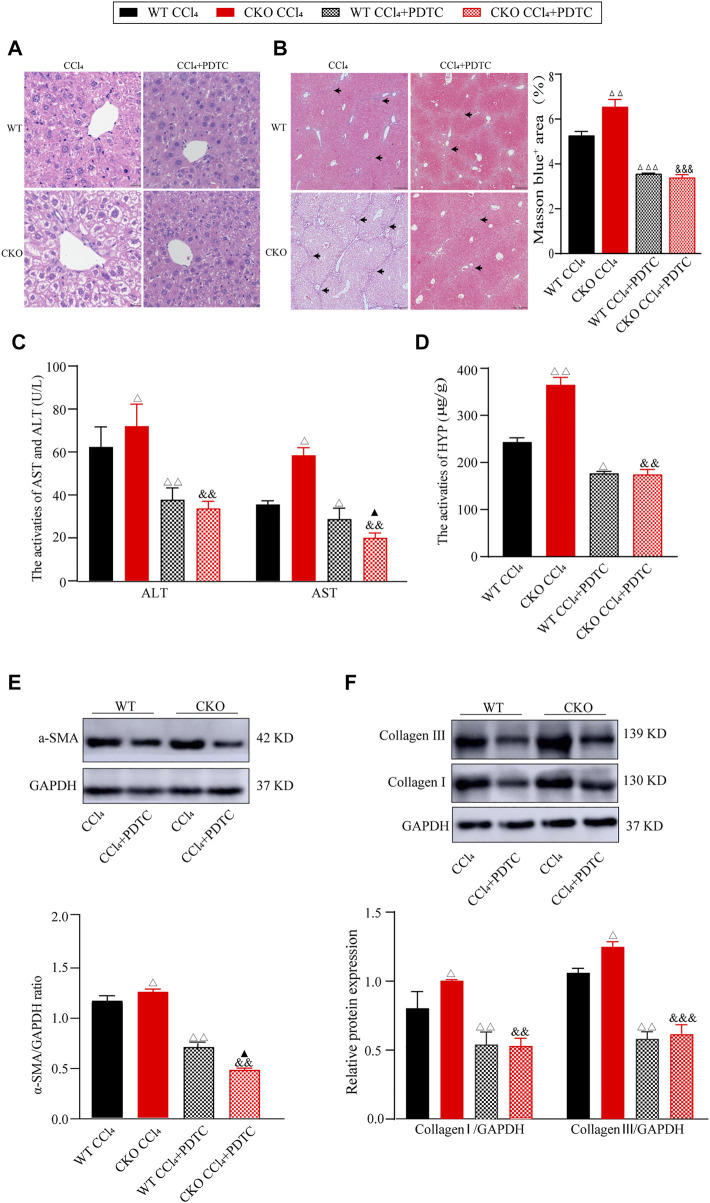
PDTC partially reverses the effects induced by PEBP4 knockout. **(A)** Histological changes of liver tissues by HE staining (×200 magnification). **(B)** Collagen deposition of liver tissues by Masson staining (×100 magnification) and its quantitative analysis. Arrowhead indicates accumulated collagen. **(C)** The AST and ALT activities in the serum of mice (*n* = 6). **(D)** The HYP activity in the serum of mice (*n* = 6). **(E)** The protein expression of α-SMA was detected by western blotting (*n* = 3). **(F)** The protein expressions of collagen I and collagen III were detected by western blotting (*n* = 3). Data are expressed as mean ± SD. ^Δ^ *P* < 0.05, ^ΔΔ^ *P* < 0.01 and ^ΔΔΔ^ *P* < 0.001 vs. the WT CCl4 group. ^&&^ *p* < 0.01 and ^&&&^ *p* < 0.001 vs. the CKO CCl4 group. ^▲^ *p* < 0.05 vs. the WT CCl4+PDTC group (ANOVA Test).

To further investigate the mediation of the NF-κB pathway in the deterioration effect of PEBP4 deletion on liver fibrosis, we firstly examined the effect of PDTC, an NF-κB pathway inhibitor, on liver fibrosis induced by CCl4. Histological analysis showed that PDTC pretreatment alleviated the severity of liver fibrosis compared to CCl4 control groups (Figures 4A,B). Likewise, compared with CCl4 control groups, PDTC treatment resulted in a decline of serum ALT and AST activities both in the WT and CKO mice (Figure 4C). Moreover, in PDTC groups, serum AST activity was lower in the CKO mice in comparison with the WT mice (*p* < 0.05). Intriguingly, the statistical difference in ALT activity between the WT PDTC and CKO PDTC groups was not shown. PDTC treatment also led to decreased HYP activity and reduced expression of ECM proteins including α-SMA, collagen I and collagen III in both CCl4-treated WT and CKO mice (Figures 4D–F). In comparison with WT PDTC group, the expression of α-SMA showed an expected decrease in CKO PDTC group (*p* < 0.05), while HYP activity and the expression of collagen I and collagen III had no statistically significant differences between the two groups.

**FIGURE 5:**
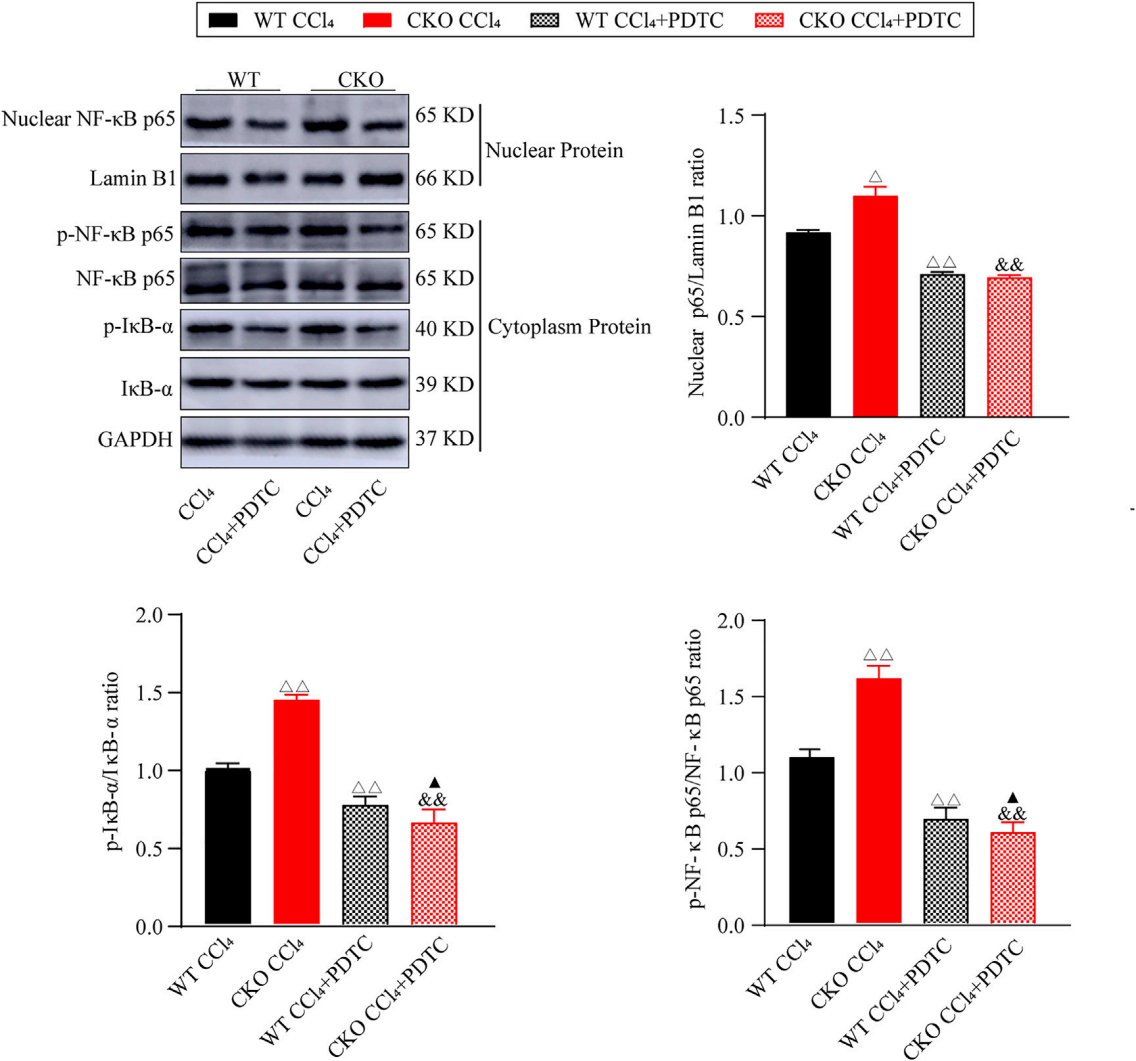
Effects of PDTC on the NF-κB pathway in liver fibrosis. Protein expressions of nuclear NF-κB p65, p-NF-κB p65 and p-IκB-α were measured by western blotting. Data are expressed as mean ± SD. ^Δ^ *P* < 0.05 and ^ΔΔ^ *P* < 0.01 vs. the WT CCl4 group. ^&&^ *p* < 0.01 vs. the CKO CCl4 group. ^▲^ *p* < 0.05 vs. the WT CCl4+PDTC group (ANOVA Test).

Furthermore, we investigated the effect of PDTC on the NF-κB signaling pathway in the fibrotic changes induced by CCl4. As expected, western blotting assays showed that PDTC suppressed nuclear NF-κB expression, and the phosphorylation of IκB-α and NF-κB p65 when compared with single CCl4-treated groups (Figure 5, *p* < 0.01). Between the two experimental groups, IκB-α and NF-κB p65 phosphorylation was reduced more significantly in CKO mice (*p* < 0.05), whereas the difference in nuclear NF-κB expression was not statistically significant. Taken together, these results indicate that the effect of PEBP4 knockout on liver fibrosis might be partly mediated by the NF-κB signaling pathway.

## Discussion

The pathophysiology of liver fibrosis has become a research hotspot in recent decades. Hepatic fibrosis is mainly caused by toxic injury or cholestatic injury. During the fibrogenesis, HSC activation concomitant with ECM secretion and accumulation is considered as an important alternation of liver fibrosis (Kisseleva and Brenner, 2021). However, the underlying mechanism of this alternation has not been fully understood. In the current study, we firstly used a CCl4-induced fibrotic model, and identified that PEBP4 was downregulated in the liver of mice treated with CCl4, implying a potential involvement of PEBP4 in hepatic fibrosis (Figure 1A). To verify our hypothesis, hepatocyte-specific PEBP4 knockout mice was constructed (Figures 1B–E). PEBP4 knockout resulted in the abnormalities in hepatocytes and hepatic lobular structure, and more collagen accumulation in the interstitial tissues of liver (Figures 2A,B). Exacerbation of liver fibrosis based on PEBP4 knockout was further recognized by increased activities of ALT, AST and HYP, whose higher activities represent liver injury and fibrosis (Figures 2C,D). Increased levels of α-SMA, collagen I and collagen III were detected as well in PEBP4 knockout fibrotic mice (Figures 2E,F), suggesting that loss of PEBP4 is accountable for intensified collagen deposition.

Previous studies on PEBP4 revealed its anti-apoptotic and pro-metastatic roles in cancers (Li et al., 2014). Although most PEBP-related studies focused on various types of cancer cells, PEBP was found to be a potential biomarker in acute liver failure (ALF) (Lv et al., 2007). Based on both animal study and detection of human PEBP levels, plasma PEBP levels were found to be elevated in ALF, suggesting a possible correlation between PEBP and liver diseases. However, there has been no report regarding the role of PEBP4 in liver diseases. Although PEBP4 was reported to promote cancer development, our findings suggested that PEBP4 unexpectedly had beneficial effects in liver fibrosis. Reasons for this inconformity might be that the functions of PEBP4 may be performed *via* different signaling pathways in liver fibrosis and cancer, in addition, the effect of PEBP4 is related to its expression level. Some studies have demonstrated that PEBP4 modulates multiple signaling pathways. For instance, overexpression of PEBP4 results in the PI3K/Akt and SHH pathways activation, whereas PEBP4 silencing activates the ERK and MAPKs pathways in lung squamous cell carcinoma (Yu et al., 2020). In this study, PEBP4 was identified to inhibit the NF-κB pathway. In this report, we found that loss of PEBP4 promoted the activation of NF-κB pathway, as characterized by upregulating the expression of nuclear NF-κB p65 and increasing the phosphorylation of IκB-α and NF-κB p65 (Figure 3), indicating a negative correlation between PEBP4 and the NF-κB pathway. Moreover, our results indicated that PEBP4 suppressed the phosphorylation of key molecules in the NF-κB signaling, specifically the phosphorylation of IκB-α and NF-κB p65, which enables fewer p65/p50 complex to enter into cell nucleus. Likewise, Raf kinase inhibitor protein (RKIP), another member of the PEBP family, was found to inhibit the phosphorylation of NF-κB pathway (Su et al., 2017). These results are consistent with our findings. Studies have shown that NF-κB signaling is involved in hepatic injury, fibrosis, and hepatocellular carcinoma (HCC) (Luedde and Schwabe, 2011). Other studies have found that inhibiting NF-κB pathway *via* different ways alleviates liver fibrosis and this function is mainly achieved by suppressing HSC activation (Liu et al., 2020; Xia et al., 2020), implying that NF-κB inhibition leads to HSC quiescence, thereby preventing liver fibrosis development.

Nonetheless, the present study surprisingly found that PDTC treatment only rescued partial features of liver fibrosis, namely the increase in the ALT and AST activities, and α-SMA expression, in CKO mice with CCl4 treatment, while HYP activity and collagen I and III levels showed no statistically significant difference in these mice after PDTC treatment. The discrepancy in the results indicates that the protective role of PEBP4 in liver fibrosis might be mediated by the NF-κB signaling, but also through other molecular pathways. The status of NF-κB signaling modulated by PEBP4 could be similar to that regulated by microRNA-378, which activated NF-κB by targeting *Prkag2*, promoting renal fibrosis (Zhang et al., 2019). Besides, angiotensin II (Ang II) can increase the level of miR-103a-3p, contributing to the over-activation of NF-κB/p65, and consequently causes renal inflammation and fibrosis (Lu et al., 2019).

In conclusion, our research proved that PEBP4 can alleviate liver fibrosis and partly suppress the NF-κB pathway. However, this study has some limitations that need to be explored in the future. For instance, the cell types that PEBP4 might act on remain undiscovered. Whether PEBP4 affects liver fibrosis *via* the suppression of HSC activation is worth investigating. Additionally, it would be better if we detect more downstream targets of the NF-κB and employ the model of PEBP4 overexpression to clarify the function of PEBP4 on liver fibrosis in our future studies. At any rate, the present findings highlight the protective role of PEBP4 in hepatic fibrosis and its potential molecular mechanism, providing a foundation for the clarification of its exact role in liver fibrosis.

## Data Availability

The original contributions presented in the study are included in the article/Supplementary Material, further inquiries can be directed to the corresponding authors.

## Glossary

Alb: albumin
Ang II: angiotensin II
ALF: acute liver failure
ALT: alanine aminotransferase
ANOVA: analysis of variance
α-SMA: α-smooth muscle actin
AST: aspartate aminotransferase
CCl4: carbon tetrachloride
CKO: conditional knockout
ECM: extracellular matrix
ERK1/2: extracellular signal-regulated kinase 1/2
GAPDH: glyceraldehyde-3-phosphate dehydrogenase
HCC: hepatocellular carcinoma
HE: hematoxylin and eosin
HSC: hepatic stellate cell
HYP: hydroxyproline
IκB-α: NF-κB inhibitor-α
MAPK: mitogen-activated protein kinase
NF-κB: nuclear factor-κappa B
NSCLC: non-small cell lung cancer
PCR: polymerase chain reaction
PDTC: pyrrolidine dithiocarbamate
PEBP4: phosphatidylethanolamine-binding protein 4
PI3K: phosphoinositide 3-kinase
PVDF: polyvinylidene fluoride
RKIP: Raf kinase inhibitor protein
SD: standard deviation
SDS-PAGE: sodium dodecyl sulfate polyacrylamide gel electrophoresis
SHH: sonic hedgehog
TNF-α: tumor necrosis factor-α
WT: wild type
